# Extraction and Identification of Three New *Urechis unicinctus* Visceral Peptides and Their Antioxidant Activity

**DOI:** 10.3390/md20050293

**Published:** 2022-04-27

**Authors:** Jingjing Li, Jiajun Lu, Charles Asakiya, Kunlun Huang, Xiuzhi Zhou, Qingliang Liu, Xiaoyun He

**Affiliations:** 1 Key Laboratory of Precision Nutrition and Food Quality, Ministry of Education, College of Food Science and Nutritional Engineering, China Agricultural University, Beijing 100083, China; 17861120538@163.com (J.L.); 17768116970@163.com (J.L.); asakiya@cau.edu.cn (C.A.); huangkl@cau.edu.cn (K.H.); 2 Key Laboratory of Safety Assessment of Genetically Modified Organism (Food Safety), The Ministry of Agriculture and Rural Affairs of the P.R. China, Beijing 100083, China; 3 Shandong Baier Testing Corp., Ltd., Weifang 261061, China; 18764613510@163.com (X.Z.); 13666361630@163.com (Q.L.)

**Keywords:** internal organs of *Urechis unicinctus*, ultrasound-assisted enzymatic hydrolysis, polypeptide, antioxidant activity

## Abstract

The viscera of *Urechis unicinctus* with polypeptides, fatty acids, and amino acids are usually discarded during processing to food. In order to improve the utilization value of the viscera of *Urechis unicinctus* and avoid resource waste, antioxidant polypeptides were isolated from the viscera of *Urechis unicinctus*. First, a protein hydrolysate of *Urechis unicinctus* (UUPH) was prepared by ultrasonic-assisted enzymatic hydrolysis, and the degree of hydrolysis was as high as 79.32%. Subsequently, three new antioxidant peptides (P1, P2, and P3) were purified from UUPH using ultrafiltration and chromatography, and their amino acid sequences were identified as VTSALVGPR, IGLGDEGLRR, TKIRNEISDLNER, respectively. Then, the antioxidant activity of the polypeptide was predicted by the structure–activity relationship and finally verified by experiments on eukaryotic cells. The P1 peptide exhibited the strongest antioxidant activity among these three antioxidant peptides. Furthermore, P1, P2, and P3 have no toxic effect on RAW264.7 cells at the concentration of 0.01~2 mg/mL and can protect RAW264.7 cells from H_2_O_2_-induced oxidative damage in a concentration-dependent manner. These results suggested that these three new antioxidant peptides were isolated from the viscera of *Urechis unicinctus*, especially the P1 peptide, which might serve as potential antioxidants applied in health-derived food or beverages. This study further developed a new use of the by-product of *Urechis unicinctus*, which improved the comprehensive utilization of marine biological resources.

## 1. Introduction

*Urechis unicinctus*, also known as “sea intestine”, belongs to echiurioidea, echiurida, xenopneusta, and urechidae [1,2]. Studies have shown that *Urechis unicinctus* is rich in multiple substances such as polypeptides, fatty acids, amino acids [3], and crude protein accounts for about 22.84% of the total. The protein can be hydrolyzed into bioactive polypeptides, which have antihypertensive effects, lowering blood sugar and antimicrobial and antioxidant activity [4,5,6]. At present, the extraction of *Urechis unicinctus* polypeptide is mainly concentrated in the body’s wall muscle [7,8], and its internal organs are directly thrown away as waste, which not only causes environmental pollution but also leads to a waste of resources. Therefore, polypeptides were extracted from the viscera of *Urechis unicinctus* in this study.

Antioxidants alleviate the damage caused by oxidative stress by preventing the formation of free radicals, scavenging free radicals, and reactive oxygen species (ROS), activating the antioxidant defense system, and performing functional repair of cells with ROS-induced damage [9,10,11,12]. Many studies have shown that oxidative damage caused by free radicals or oxidative stress in the body is closely related to aging, Alzheimer’s disease, Parkinson’s disease, and cardiovascular disease [11]. Therefore, looking for potential natural antioxidants to replace synthetic products has become a research hotspot, especially some fish-processing by-products. Hydrolyzed protein has become a hot topic in pharmacy and health food [13]. Studies have shown that chickpea proteolytic peptides have chelating iron activity [14], and the use of vegetable protein hydrolysates as food additives is already allowed in the United States. The viscera of *Urechis unicinctus* are inedible and rich in protein, so it is a good raw material for the industrial production of active peptides.

Consequently, this study isolated and identified three novel antioxidant peptides from UUPH using ultrasonic-assisted enzymatic hydrolysis. In addition, the LC-MS/MS method was used to identify amino acid sequences of peptides. Then, molecular docking was used to verify the antioxidant mechanism of peptides. Finally, the cytoprotective effect of peptides on H_2_O_2_-damaged RAW264.7 cells was determined.

## 2. Results and Discussion

### 2.1. Degree of Hydrolysis (DH)

In this study, ultrasound-assisted enzymatic technology was used for the enzymolysis of *Urechis unicinctus* protein. The final DH of UUPH measured was 79.32%. On the other hand, Zhang et al. [15] did not use ultrasound-assisted enzymolysis technology to hydrolyze *Urechis unicinctus* protein, and its DH was only 17.8%. The results show that the ultrasound-assisted enzymatic hydrolysis process can significantly improve the enzymatic hydrolysis reaction speed of *Urechis unicinctus* protein, which is consistent with the effect of ultrasound reported in the literature. In addition, ultrasound plays a synergistic role by reducing steric hindrance and making the substrate (specific peptide sequences) more accessible [16].

### 2.2. Ultrafiltration and Determination of Antioxidant Activity before and after Simulated Gastrointestinal Digestion

Ultrafiltration is often used as the first step of isolation and purification to realize the crude separation of protein hydrolysates according to the molecular weight obtained from the desired antioxidative components [17]. The UUPH-1 was divided into three parts by the ultrafiltration tube: UUPH-2 (>30 kDa), UUPH-3 (10–30 kDa), UUPH-4 (<10 kDa).

**Figure 1:**
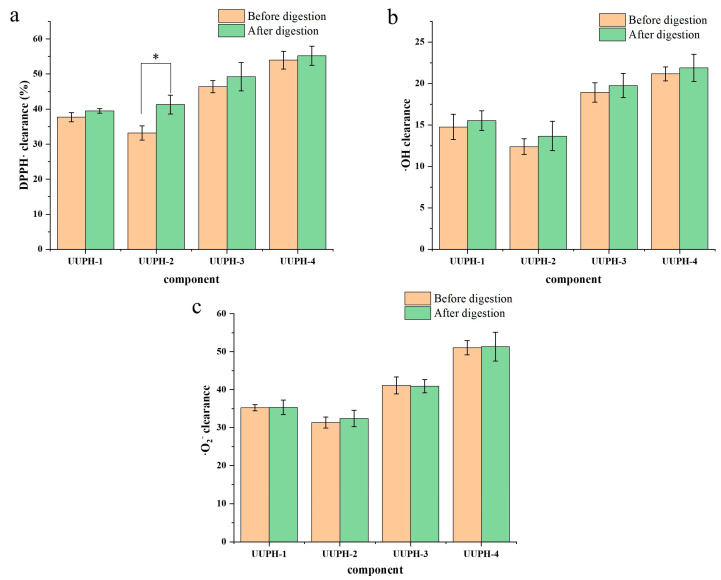
Determination of the antioxidant activity of each component. (**a**) DPPH clearance. (**b**) OH clearance. (**c**) ·O_2_ clearance. Note: * indicates *p* value < 0.05.

Activities of the compounds were evaluated using their DPPH·scavenging activity, hydroxyl radicals (·OH) scavenging activity, and superoxide anion free radical (·O_2_^−^) scavenging activity. As shown in Figure 1, at the concentration of 0.5 mg/mL, the antioxidant activity was UUPH-4 > UUPH-3 > UUPH-1 > UUPH-2 before digestion. The antioxidant activity of UUPH-1 was slightly higher than that of UUPH-2, which may be due to it containing a small amount of UUPH-1 and UUPH-4. The results showed that the antioxidant activity of compounds with smaller molecular weight (MW) is generally better than that of compounds with larger average MWs. The results were consistent with those of other studies. For example, Xing et al. [18] isolated the antioxidant peptide from ham and measured the antioxidant activity of each component. The results showed that the component with MW < 3 kDa had the highest antioxidant activity; Canabady Rochelle et al. [19] and Wang et al. [20] have shown that low molecular weight peptides can terminate free radical chain reactions and chelate transition metal ions better than high molecular weight peptides. After digestion, the antioxidant activity of each component changed, but the antioxidant activity of UUPH-4 was still the highest. Therefore, UUPH-4 components were collected for further study.

### 2.3. Gel-Filtration Chromatography

**Figure 2:**
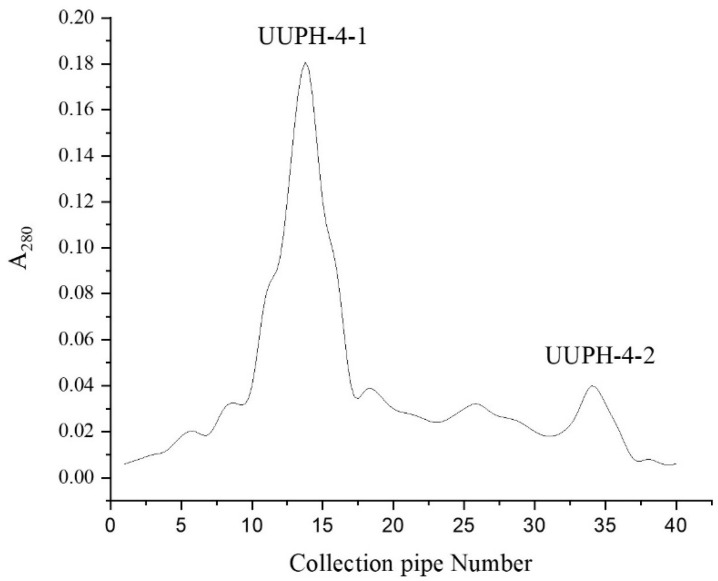
Sephadex G-25 gel column chromatography results.

Sephadex G-25 gel-filtration chromatography is an effective method for separating compounds according to the MW, widely used to isolate peptides in protein hydrolysates [21]. As shown in Figure 2, the UUPH-4 was separated on Sephadex G-25 to yield two fractions. The DPPH scavenging rate of UUPH-4-2 was 29.8%. On the other hand, the DPPH scavenging rates of UUPH-4-1 reached 58.5%, significantly higher than UUPH-4-2 (the protein concentration was 0.5 mg/mL). This result is consistent with previous reports that smaller MW of peptides have better antioxidant activities [22]. This result further proves that MW is a key factor affecting the antioxidant capacity of peptides [23].

### 2.4. Identification of Peptide Sequences

**Figure 3:**
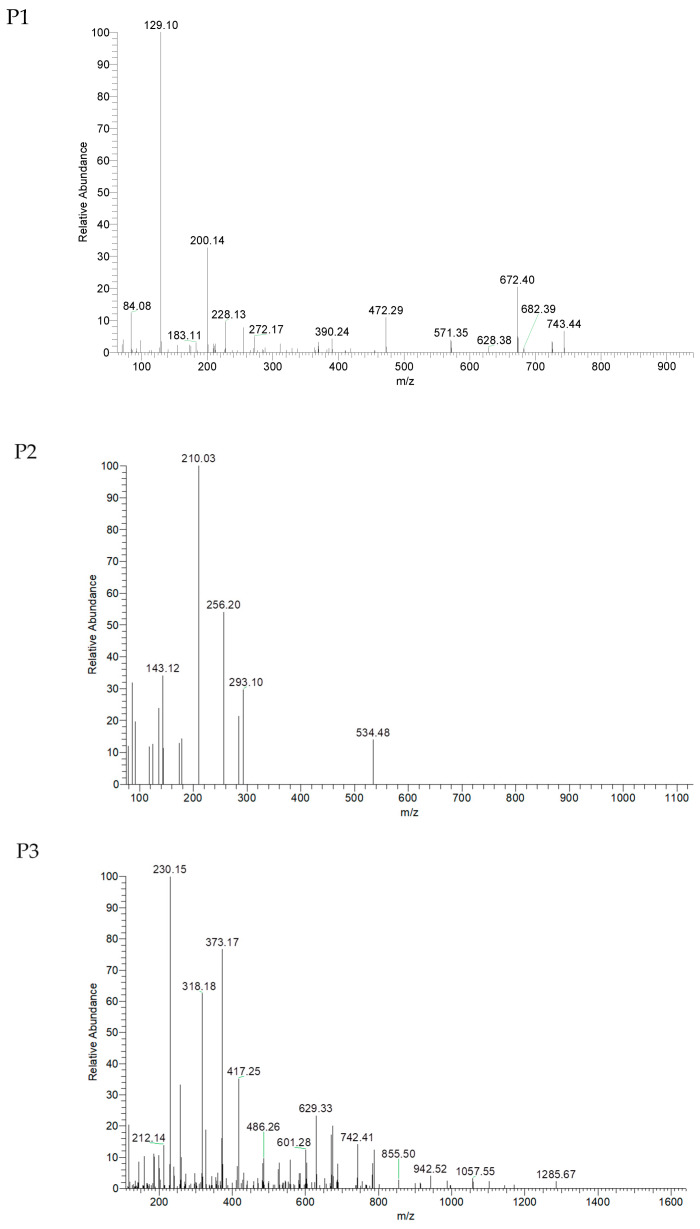
LC-MS/MS spectrogram analysis of peptides (P1, P2, and P3).

As shown in Figure 3, UUPH-4-1 was isolated by LC-MS/MS to afford three main peptides, named P1, P2, and P3. The sequence of each of the peptides was determined using the de nava algorithm in mascot software (version 2.2, Matrix Science, Boston, MA, USA). As shown in Table 1, P1, P2, and P3 sequences were identified as VTSALVGPR, IGLGDEGLRR, and TKIRNEISDLNER, respectively. Their MWs were 898.5, 1084.6, and 1586.8 Da. P1, P2, and P3 peptides contain amino acids related to antioxidant activity such as Pro, Gly, Ala, Val, or Leu [24].

### 2.5. The Structure–Activity Relationship

At present, there are two main research methods on the structure–activity relationship of antioxidant peptides; one is induction and the other is bioinformatics; that is, the molecular model of antioxidant peptides is constructed by a computer, and the interaction between antioxidant peptides and receptors is explored by molecular docking.

#### 2.5.1. Induction

Firstly, the antioxidant activity of three peptides was predicted by induction. Many studies have proved that the antioxidant properties of peptides are related to their MW, composition, secondary structure, and hydrophobicity [25,26].

Studies found that the antioxidant activity of antioxidant peptides was negatively correlated with the MW of peptides, and the MW of most antioxidant peptides was less than 3 kDa [27]. As shown in Table 1, the MWs of P1, P2, and P3 were below 3 kDa, and the MW of P1 was the lowest. Therefore, it can be predicted from the MW that the antioxidant activity of P1 was the highest and P3 was the lowest.

In terms of amino acid composition, studies suggested that antioxidant peptides generally contain the following five amino acids: Pro, Gly, Ala, Val, and Leu [24]. The higher the total proportion of these five amino acids, the higher the antioxidant activity. It can be inferred from Table 2 that P1 had the highest antioxidant activity and P3 had the lowest.

**Figure 4:**
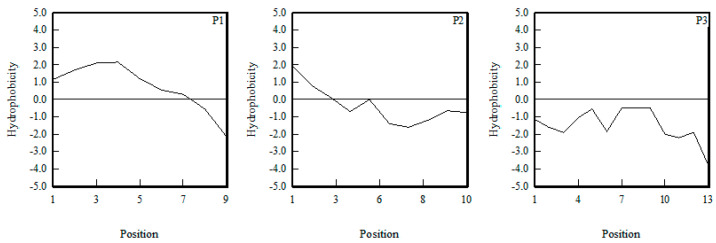
Hydrophobicity analysis of P1, P2, and P3.

Hydrophobic amino acid residues facilitate the interaction between antioxidant peptides and fat-soluble free radicals; thereby improving the ability to inhibit lipid peroxidation [25]. For example, in the study of oyster protein hydrolysates, Wang et al. found that the hydrophobic peptides can form complexes with zinc ions [28]. At the same time, hydrophobic amino acid residues located at the C-terminal or N-terminal contributed to the activities of antioxidant peptides. As shown in Figure 4, P1 had the greatest hydrophobicity, and the hydrophobic amino acid residues were mainly located at the N-end. P3 had the least hydrophobicity and had no hydrophobic amino acid residue. Therefore, in terms of hydrophobicity, P1 had the highest antioxidant activity and P3 had the lowest antioxidant activity.

**Figure 5:**
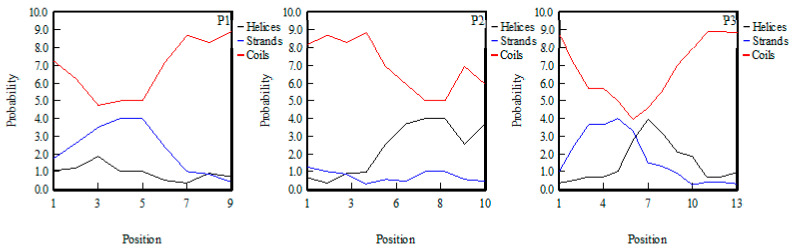
Results of secondary structure analysis of P1, P2, and P3.

Previous research suggested that the secondary structure of antioxidant peptides had more β-folding and irregular coils and less α-helix, which may be due to the fact that β- folding and irregular coils can expose more active sites of the peptides. At the same time, β-folding can also increase the structural stability of antioxidant peptides, so peptides are not easily affected by the environment [24]. As shown in Figure 5, P1 had the largest proportion of β-folding and irregular crimping structure and the least α-helix, followed by P3, and finally P2. Therefore, from the secondary structure, the antioxidant activity of P1 was the highest and P2 was the lowest.

Finally, in terms of MW, amino acid composition, hydrophobicity, and secondary structure, the order of antioxidant activity of P1, P2, and P3 was P1 > P2 > P3.

#### 2.5.2. Molecular Docking

The Keap1-Nrf2 signal pathway is a classical signaling pathway of antioxidant damage in the human body [29]. The starting point of signaling pathway activation is the dissociation of Keap1 and Nrf2. The most important dissociation mode is competitive inhibition; that is, foreign molecules act on the binding region of keap1-Nrf2 to enhance the dissociation of Nrf2 and, in turn, promote the antioxidant damage of body cells.

**Figure 6:**
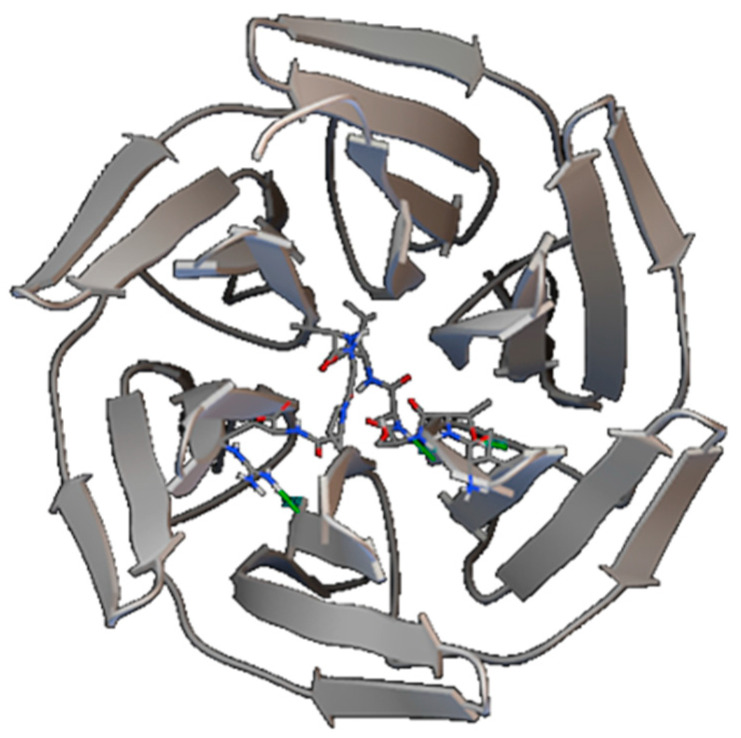
Three-dimensional structure of P1 combined with the kelch region.

The docking study was carried out by the kelch region bound by keap1-Nrf2 as the target. The results showed that only the P1 peptide interacted with the kelch region of Keap1 protein, the minimum binding free energy was −4.01 kcal/mol, and the main interaction between P1 and the kelch region was hydrogen bonding. The action sites shown in Figure 6 are Thr2 of P1 and Gly423 of the kelch region; Ala4 of P1 and Val420 of the kelch region; and Arg9 of P1 and Asn469 of the kelch region, respectively. The results showed that among the three peptides, the P1 peptide might play an antioxidant role via activation of the keap1-Nrf2 pathway, and the other two peptides may play an antioxidant role through other mechanisms.

### 2.6. Cytoprotective Activity of Peptide on H_2_O_2_-Damaged RAW264.7 Cells

#### 2.6.1. Cytotoxicity

**Figure 7:**
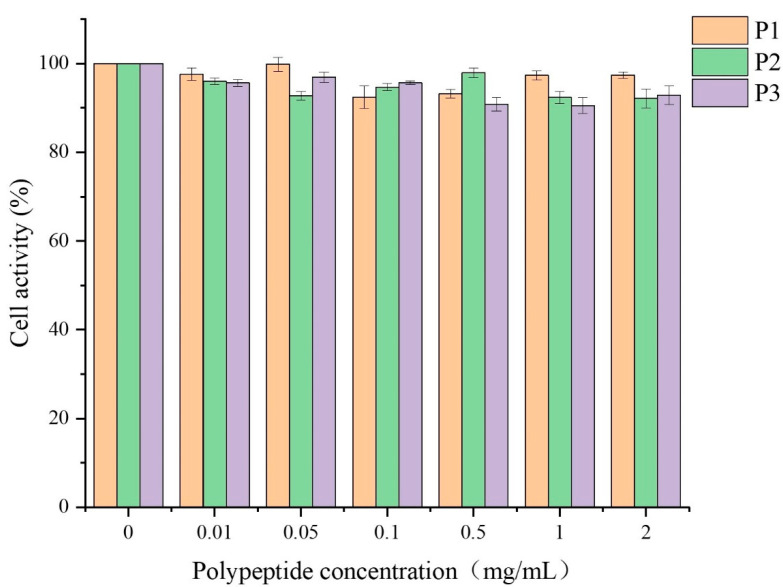
Effect of antioxidant peptides on the activity of RAW264.7 cells.

Cytotoxic effects of the three isolated peptides (P1, P2, and P3) at the concentration of 0.01–2 mg/mL were determined in RAW264.7 cells by CCK-8 assay. As shown in Figure 7, three peptides exhibited no significant effect on the viability of RAW264.7 cells compared to the blank control group at the test concentration. The result indicated that the three peptides could serve as potential antioxidants applied to food.

#### 2.6.2. Protection of Peptide on H_2_O_2_-Induced Oxidative Damage RAW264.7 Cells

**Figure 8:**
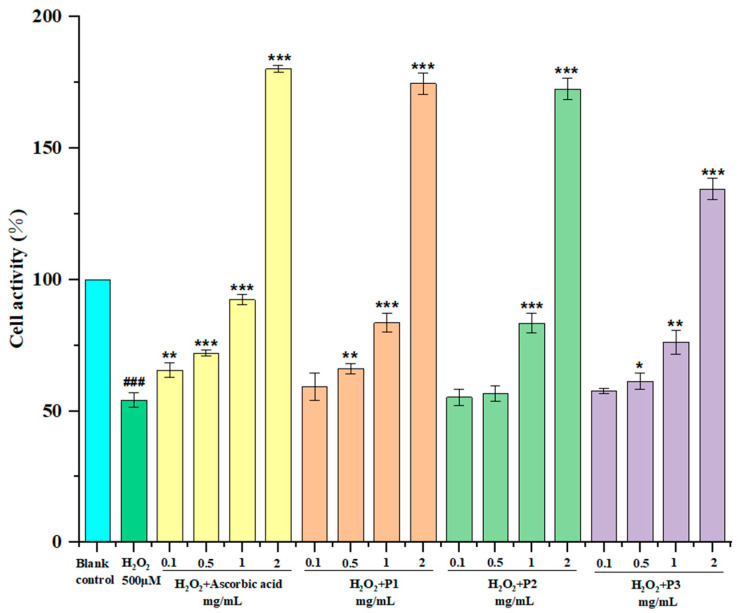
Protective effects of three isolated peptides (P1, P2, and P3) on H_2_O_2_-induced oxidative damage in RAW264.7 cells at concentrations of 0.1, 0.5, 1.0, and 2.0 mg/mL. Ascorbic acid was used as the positive control. The data are presented as the mean ± SD (*n* = 3). ^###^ *p* < 0.001 versus the blank control group. * *p* < 0.05 versus the H_2_O_2_ treated group. ** *p* < 0.01 versus the H_2_O_2_ treated group. *** p < 0.001 versus the H_2_O_2_ treated group.

The RAW264.7 cells were treated for 24 h with the peptides and ascorbic acid; then, incubated with hydrogen peroxide for another 24 h. Figure 8 shows the influences of three peptides and ascorbic acid on H_2_O_2_-induced oxidative damage RAW264.7 cells. Three isolated peptides treated groups were gradually increased in a concentration-dependent manner. The P1 treated group increased the RAW264.7 cell viability from 54.52% to 55.23%, 66.18%, and 83.62% at the concentrations of 0.1, 0.5, and 1 mg/mL, respectively. Moreover, cells treated with 2 mg/mL of P1, P2, P3, and ascorbic acid restored cell viability up to 174.6%, 172.5%, 134.5%, and 180.2% (the 100% viability value refers to untreated control cells), which strongly protect H_2_O_2_-induced oxidative damage of RAW264.7 cells and promote cell proliferation, especially at the high concentrations. This is consistent with the report of Li et al. [30]. Compared with Figure 7, cells treated with 2 mg/mL peptide for 12 h did not promote cell proliferation, which may be due to the insensitive absorption of peptides. In general, the antioxidant activity of P1 was the best, which was close to ascorbic acid, followed by P2.

## 3. Materials and Methods

### 3.1. Materials

*Urechis unicinctus* was provided by the Yantai breeding base. Papain, trypsin, alcalase, pepsin, and compound protease were purchased from Anning Dongheng Huadao Biotechnology Co. Ltd. (Nanning, China). Superoxide anion free radical assay kit was purchased from the Beijing Solabao Technology Co. Ltd. (Beijing, China). Sephadex G-25 was purchased from Shanghai Yuanye Biotechnology Co., Ltd. (Shanghai China). DPPH was purchased from Sigma Corporation (St. Louis, MO, USA). RAW264.7 cells were purchased from Shanghai Biyuntian Biotechnology Co. Ltd. (Shanghai, China).

### 3.2. Preparation of Protein Hydrolysate from Urechis unicinctus Viscera (UUPH)

After degreasing with ethyl acetate, the viscera of *Urechis unicinctus* were dried and crushed. The visceral degreasing powder was prepared into a solution by adding water according to the material–liquid ratio of 27:1 (mL/g), adjusting pH = 7. UUPH samples were prepared by enzymatic hydrolysis using papain, trypsin, and alkaline protease at ultrasonic power of 675 W for 33 min; then hydrolyzed in a water bath at 50.0 °C for 2.0 h (the addition of papain was 0.6%, trypsin was 1.44%, and alkaline protease was 0.96%). After the reaction, the mixture was heated in 95 °C water for 15 min to inactivate the enzyme. Next, the hydrolysate was centrifuged at 5000× *g* for 10 min, and the supernatant was added in 2% granular activated carbon to decolorize at 60 °C for 60 min. The filtrate was collected by vacuum suction filtration, and subsequently, the filtrate was freeze-dried to obtain powdery material, which was stored at 20 °C for further use. The ultrasonic-assisted enzymatic hydrolysis conditions and enzymatic hydrolysis parameters in this experiment were optimized.

### 3.3. Degree of Hydrolysis (DH)

The DH of UUPH was calculated based on formaldehyde titration in GB 5009.235-2016 [31] using the following formula:

Ammonia nitrogen content (g/100 mL) = C × (V_1_ − V_2_) × 0.014 × 100

where C denotes the concentration of NaOH solution (mol/L), V_1_ stands for NaOH volume (mL), and V_2_ is the volume of NaOH (mL) in the blank.

The degree of hydrolysis was calculated via the following equation:

DH (%) = m/M × 100

where m is the mass of ammonia nitrogen in UUPH and M stands for the total nitrogen mass in the sample.

### 3.4. Isolation and Purification of UUPH

#### 3.4.1. Ultrafiltration Separation

The UUPH fraction was intercepted through an ultrafiltration tube using molecular weight cut-offs of 10 and 30 kDa. UUPH was fractionated into four fractions, including UUPH-1, UUPH-2 (>30 kDa), UUPH-3 (10–30 kDa), and UUPH-4 (<10 kDa).

#### 3.4.2. Simulation of Gastrointestinal Digestion In Vivo

Freeze-dried powders of 4 components were prepared in 0.5 mg/mL protein solution. Pepsin was added at 2%, the pH value was adjusted to 2 with 1 mol/L HCl solution, and the system temperature was kept at 37 °C for enzymatic hydrolysis for 2 h. Then, the pH value was adjusted to 7 with the NaOH solution (1 mol/L), and the trypsin was added at 4%. The system’s temperature was kept at 37 °C for 4 h, and then the antioxidant activity of each component after simulated digestion was determined. See step 3.7 for the antioxidant activity determination. The components with the highest antioxidant activity were selected for subsequent separation and purification.

#### 3.4.3. Gel Filtration Chromatography (GFC)

The UUPH-4 fraction was further separated into two fractions (UUPH-4-1 and UUPH-4-2) using a Sephadex G-25 column. The elution conditions were as follows: ultrapure water and the flow rate was 0.5 mL/min. The elution flow rate was controlled at 0.5 mL/min. This eluted solution was collected every 5 min and evaluated at 280 nm. Taking the DPPH·scavenging activity as an indicator, the component with the strongest hydroxyl radical scavenging rate was selected for the next separation.

### 3.5. Peptide Identification by LC-MS/MS

Desalted UUPH aqueous solutions were filtered using a 0.22 μm membrane and the extract (10 μL) was injected into the chromatographic column (Acclaim PePmap 100 C18 traps) through an auto-sampler. The mobile phase was water with 0.1% formic acid (solvent A) and acetonitrile with 0.1% acetic acid (solvent B); the flow rate was 0.4 μL/min. The gradient elution of 0–6 min/5–8% B, 6–40 min/8–30% B, 40–45 min/30–60% B, 45–48 min/60–80% B, 48–56 min/80% B, 56–58 min/80–5% B, 58–65 min/5% B was applied. The amino acid sequence of peptides was determined using LC-MS/MS. The positive ion mode used for analysis with an ion source voltage was 1800 V and the capillary temperature was 360 °C. Primary mass spectrometry parameters: the scanning range was 350–20,000 DA and the scanning resolution was 70,000; secondary mass spectrometry parameters: the scanning range depends on the mass charge ratio of primary parent ions and the scanning resolution was 17,500.

The mass spectra were retrieved by mascot software (version 2.2) and analyzed using the de nava algorithm.

### 3.6. Molecular Modeling

The three-dimensional structure of the peptide was downloaded from the PEP-FOLD3 (https://bioserv.rpbs.univ-paris-diderot.fr/services/PEP-FOLD3/, 10 December 2020) and used as a ligand. The PDB file of the kelch region of the Keap1 protein (PDB ID: 1u6d) was downloaded using PDB (http://www.rcsb.org/, 10 December 2020) and used as a receptor. All the water molecules were removed, and the construction structures of the peptide were optimized using AutoDockTools software. The AutoGrid and AutoDock were run, the relationship between the polypeptide and the Keap1-Nrf2 signal pathway was judged according to the binding free energy of the receptor and ligand. The smaller binding free energy indicated that the polypeptide was likely to activate the Keap1-Nrf2 signal pathway and play an antioxidant role.

### 3.7. Antioxidant Activity of the Peptides

#### 3.7.1. DPPH· Scavenging Activity

The DPPH· scavenging activity was determined as described by Song et al. [32]. First, 0.1 mmol/L DPPH·solution was prepared with anhydrous ethanol. Then, 2 mL samples were mixed with 2 mL of fresh DPPH solution. The mixture was incubated for 30 min in a dark environment. The absorbance was measured at 517 nm. Subsequently, anhydrous ethanol and DPPH· solution were used as the blank and control, respectively. The DPPH scavenging rate was calculated via the following equation:

$$\mathrm{DPPH}\;\mathrm{scavenging}\;\mathrm{rate}\;(\%)=(1-\frac{A_{1}-A_{2}}{A_{0}})\times 100;$$

where *A*_1_ is the sample absorbance, *A*_2_ is the control absorbance, and *A*_0_ is the blank absorbance.

#### 3.7.2. Hydroxyl Radicals (·OH) Scavenging Activity

The·OH scavenging activity was determined as described by [33]. First, 9 mmol/L FeSO_4_ solution was prepared with ultrapure water, and 9 mmol/L salicylic acid-ethanol solution was prepared with anhydrous ethanol. Then, 0.1 mL of 30% H_2_O_2_ was diluted with ultrapure water to 100 mL. Next, 1 mL of salicylic acid solution, 1 mL H_2_O_2_ solution, and 1 mL sample solution were mixed and incubated for 30 min in a dark environment. The absorbance was measured at 510 nm. Ultrapure water was used as the blank. The ·OH scavenging rate was calculated via the following equation:

$$\cdot \mathrm{OH}\;\mathrm{scavenging}\;\mathrm{rate}\;(\%)=(1-\frac{A_{1}-A_{2}}{A_{0}})\times 100$$

where *A*_1_ is the sample absorbance, *A*_2_ is the control absorbance, and *A*_0_ is the blank absorbance.

#### 3.7.3. Superoxide Anion Free Radical (·O_2_^−^) Scavenging Activity

The·O_2_^−^ scavenging assay was performed using a ·O_2_^−^ kit. In brief, the assay was performed according to the instructions accompanying the kit. The ·O_2_^−^ scavenging rate was calculated via the following equation:

$$\cdot {O_{2}}^{-}\;\mathrm{scavenging}\;\mathrm{rate}=(1-\frac{A_{1}-A_{2}}{A_{0}})\times 100$$

where *A*_1_ is the sample absorbance, *A*_2_ is the control absorbance, and *A*_0_ is the blank absorbance.

### 3.8. Cell Cytotoxicity Assay

The isolated peptides were dissolved in the DMEM medium containing 10% fetal bovine serum with the concentrations of 0, 0.01, 0.05, 0.1, 0.5, 1, and 2 mg/mL, respectively. The RW264.7 cells were grown (1.0 × 10^4^ cells/well) in a 96-well plate for 24 h. After that, RW264.7 cells were cultured at designed concentrations of peptide solution for 12 h. The cell viability was measured by the CCK-8 test. The WST-8 in the CCK8 kit can be reduced by dehydrogenases in mitochondria to produce orange yellow formazan, then can quantify the number of viable cells by colorimetry and, thus, the cell viability is detected.

### 3.9. The Cytoprotective Activity of Antioxidant Peptide on Oxidative Damaged RW264.7 Cells by H_2_O_2_

The *Urechis unicinctus* antioxidant peptide was synthesized by Beijing Zhongke Yaguang Biotechnology Co., Ltd. (Beijing, China) by solid-phase synthesis according to the sequence identification results of Section 2.4. The RW264.7 cells were grown (1.0 × 10^4^ cells/well) in a 96-well plate for 24 h. Then, the supernatant was aspirated and 190 μL of peptide sample and ascorbic acid were added into the protection groups, respectively, for incubation for 24 h, 10 μL H_2_O_2_ (500 μmol/L) was added into the damage and protection groups and sequentially incubated for 24 h. The cell viability was measured by the CCK-8 test.

### 3.10. Statistical Analysis

The data are reported as the mean ± standard deviation (SD). Origin 2019 was used to draw the graphs. Sequence analysis was performed using DNAMAN software. Autodock4 software was used to simulate the interaction between antioxidant peptides and the kelch region of the Keap1 protein in the molecular docking experiment.

## 4. Conclusions

In this study, three antioxidant peptides (P1–P3) were isolated and purified from *Urechis unicinctus* visceral protein hydrolysate using ultrasound-assisted enzymatic hydrolysis. Their sequences were VTSALVGPR, IGLGDEGLRR, and TKIRNEISDLNER, respectively. Induction, molecular docking, and protection on H_2_O_2_-induced oxidative damage RAW264.7 cells were used to evaluate the antioxidant activity of three peptides. Among them, P1 peptide exhibited the strongest antioxidant activity. These results suggested that the three peptides could be applied as potential antioxidants in health-derived food or beverages. This study developed new uses for *Urechis unicinctus* by-products and improved the comprehensive utilization of marine biological resources. In the future, we will explore how to optimize the experimental conditions to improve the yield of these active peptides. In addition, animal feeding experiments on the three isolated peptides (P1, P2, and P3) will be conducted to evaluate their antioxidant effect and antioxidant mechanism in vivo in our lab.

## Figures and Tables

**Table 1:** Retention time (RT), amino acid sequence, and molecular weight (MW) of peptides.

No.	RT (min)	Amino Acid Sequence	MW (Da)
P1	31.52	VTSALVGPR	898.5
P2	43.87	IGLGDEGLRR	1084.6
P3	56.72	TKIRNEISDLNER	1586.8

**Table 2:** Proportion of amino acid composition of P1, P2, and P3.

Amino Acids	P1	P2	P3
A(Ala)	8.84	0	0
G(Gly)	7.2	18.05	0
L(Leu)	12.57	21.03	7.27
P(Pro)	11.04	0	0
V(Val)	22.46	0	0

## Data Availability

The study did not report any data.
